# Factors affecting the microbiological quality and contamination of farm bulk milk by *Staphylococcus aureus* in dairy farms in Asella, Ethiopia

**DOI:** 10.1186/s12866-022-02746-0

**Published:** 2023-03-07

**Authors:** Abiot Deddefo, Gezahegne Mamo, Minda Asfaw, Kebede Amenu

**Affiliations:** 1 College of Agriculture and Environmental Sciences, Arsi University, P.O.Box 193, Asella, Ethiopia; 2 College of Veterinary Medicine and Agriculture, Addis Ababa University, P.O.Box 34, Bishoftu, Ethiopia; 3 Animal and Human Health Programme, International Livestock Research Institute (ILRI), P.O.Box 5689, Addis Ababa, Ethiopia

**Keywords:** Bacterial counts, Farm bulk milk, Risk factors, *Staphylococcus aureus*

## Abstract

**Background:**

The determination of the microbiological quality and safety of raw milk and the associated influencing factors at the farm level is very critical given that the quality or safety of subsequent products that are further produced depends on this. Therefore, this study aimed to determine the microbiological quality and safety of bulk milk and identify associated risk factors, and assess the presence/absence of *S. aureus* in bulk milk with potential contaminating sources in dairy farms in Asella, Ethiopia.

**Results:**

The geometric means of bacterial counts in farm bulk milk were 5.25 log cfu/ml, 3.1 log cfu/ml and 2.97 log cfu/ml for total bacterial count (TBC), coliform count (CC) and coagulase-positive staphylococci count (CPS), respectively. Of the 50 dairy farms, 66, 88, and 32% had TBC, CC and CPS counts, respectively, that exceeded the standard international limits for raw cow’s milk intended for direct human consumption. TBC tended to increase as CC increased in bulk milk (r = 0.5). In the final regression model, increased TBC, CC and the contamination of farm bulk milk by *S. aureus* were significantly associated with dirty barns, dirty cows and soiled udder and teats. TBC was higher during the rainy season than during the dry season. The reported practice of washing teats with warm water significantly decreased CC and CPS. The occurrence of *S. aureus* was significantly (*p* < 0.05) higher in bulk farm milk (42%) than in pooled udder milk (37.3%), teat swabs (22.5%), milkers’ hand swabs (18%), bulking bucket swabs (16.7%), milking container swabs (14%), and water for cleaning of udder and milkers’ hands (10%). The questionnaire survey result showed widespred raw milk consumption habits, low level of training and poor hygienic milking practices.

**Conclusions:**

This study revealed low-quality bulk farm milk with high bacterial counts and a high occurrence of *S. aureus*. This indicates the potential food safety risks due to consumption of raw milk or its products. This study suggests awareness creation to dairy farmers and the public on hygienic milk production and heat treatment of milk before consumption.

## Background

Milk is a very nutritious food in nature, playing an important role in human diets, but at the same time, it serves as an ideal medium for microbial growth, negatively affecting the health of consumers [1]. Raw milk, as it leaves the udders of healthy animals, contains very low numbers of microorganisms and is expected to be safe for human consumption. After secretion from udder, milk can be immediately contaminated by spoilage bacteria and pathogens from various sources, including animal faeces, soil, air, feed, water, bedding material, animal hide, infected udder, the exterior of udder and teats, milk containers and people [2, 3].

The quality of milk produced at the farm level depends on farm management practices, and hygienic milk production at the farm level is a key factor for protecting milk from microbial contamination and safeguarding consumers from milk-borne health risks [4]. The initial microbial load of raw milk at farm level determines the quality of products across the dairy chain [5]. For example, if the initial bacterial load is high, further heat treatments may not sufficiently eliminate them [6]. At the farm level, microbial contamination of bulk milk occurs via 3 main sources: bacterial contamination from the external surface of the udder and teats, from the surface of the milking equipment, and from mastitis organisms from within the udder [7].

Bulk milk analysis is a useful tool for evaluating milk quality and monitoring udder health status at the herd level. The hygienic quality of milk can be determined using several microbiological count methods, including total bacterial count (TBC), coliform count (CC) and coagulase-positive staphylococci count (CPS) [8]. TBC provides an estimate of the total number of aerobic bacteria present in raw milk [9] and is the most common method to evaluate the hygienic quality of raw milk at the farm level [10]. TBC is a useful indicator of the hygienic condition of the farm environment, the cow and the milking equipment [11, 12]. An increase in the TBC of bulk tanks can be related to environmental contaminants, unsanitary milk equipment, dirty udders and teats, and mastitis causing organisms [10, 13]. Temperature of storage and time since milking are also important in determining milk quality, as these factors influence the rate at which bacteria increase in number [4]. A high level of TBC (> 10^6^ cfu/ml) is associated with increased enzyme activity that can result in textural and flavour defects in raw milk and processed dairy products [14, 15].

Coliform count estimates the number of coliform bacteria in milk [16]. Coliforms in milk primarily originate from the cow’s environment. Coliform bacteria in milk are an indicator of faecal contamination, often from soiled udders and teats. High levels of CC (> 10^3^ cfu/mL) in raw milk may indicate poor environmental hygiene, poor hygienic milking practices and further handling, improperly cleaned milk equipment, contaminated water, inadequate refrigeration, or the presence of coliform mastitis [13, 17, 18]. High CC indicates possible contamination of milk by pathogenic bacteria of public health significance, such as Shiga toxin-producing *E. coli* O157:H7 [15, 19].

*Staphylococcus aureus* is a major cause of bovine mastitis [20], resulting in large economic losses due to reduced milk production and quality [21]. *S. aureus* is frequently isolated from cows with mastitis [22–25] and bulk milk [26–28] in Ethiopia. The presence of *S. aureus* in bulk milk samples indicates that infected cows are present in a herd [9]. The public health concern due to staphylococcal food poisoning is potentially serious in Ethiopia due to widespread raw milk consumption habits [29–31]. *S. aureus* produces enterotoxins that cause food poisoning when ingested in contaminated food. Milk and milk products are frequently implicated in staphylococcal food poisoning [32].

In the urban and peri-urban areas of Asella (the present study site), farmers keep mainly cross-breed dairy cows in intensive or semi-intensive management system and produce milk for sale. Except in a few milk marketing cooperatives, milk is predominantly marketed through informal channels [33, 34].

Although some research works have been conducted in different parts of the country on the microbiological quality of farm bulk milk, there is limited scientific data regarding factors that affect the microbiological quality and contamination of bulk milk by *S. aureus* in Ethiopia and particularly in the study area. Moreover, sources of farm bulk milk contamination by *S. aureus* were not well studied. Such information is crucial for the improvement of milk quality as well as safeguarding the public from milk-borne diseases. Therefore, this study aimed to determine the microbiological quality and safety of bulk milk and identify associated risk factors, and assess the presence/absence of *S. aureus* in bulk milk with potential contaminating sources in dairy farms in Asella, Ethiopia.

## Methods

### Description of study area

The study was conducted in Asella, Arsi zone, Oromia regional state, Ethiopia. Asella is located at a latitude and longitude of 7°57ˈN and 39°7ˈE, respectively. Asella is the capital of Arsi zone and is located 175 km southeast of Addis Ababa in a highland plateau region at an elevation of 2430 m above sea level. Asella is categorized as having a subtropical highland climate with an annual mean rainfall and temperature of 1100 mm and 15.47 °C, respectively. The area experiences a bimodal rainfall pattern, with a short rainy season occurring during March and April and a long rainy season extending from June to September [35]. Arsi zone, especially Asella area, has a conducive climate for rearing specialized dairy breeds [34]. The area was where the first small-scale dairy development was initiated in Ethiopia in collaboration with Swedish government [36]. According to the Central Statistical Agency [37], Arsi zone has a cattle population of 2,904,201, which is the largest from the zones of the Oromia region. The zone has 692,724 heads of cows and 154,961cross breed cows.

### Study design and sampling technique

A cross-sectional study was conducted from January 2021 to February 2022 to assess factors affecting the microbiological quality and contamination of farm bulk milk by *S. aureus* in dairy farms in Asella, Ethiopia. A total of 434 samples were collected from 50 dairy farms. Of this, 50 were farm bulk milk, 102 pooled udder milk, 50 water for cleaning of udder and hands, 50 milking container swabs, 30 bulking bucket swabs, 50 hand swabs and 102 teat swab samples. Asella was purposively selected based on milk production potential and accessibility. *Kebeles* (the smallest administrative units) were selected by the simple random sampling technique, and dairy farms or households were selected randomly within the *Kebeles*. The study animals included crossbred lactating dairy cows (Holstein-Friesian x Zebu) of all age categories kept under intensive and semi-intensive management systems. The sampling was limited to 50 farms because of limitations related to time and logistics.

### Sample collection and transportation

Raw milk samples were collected according to the National Mastitis Council milk sample collection and handling guidelines [38]. Strict aseptic procedures were followed when collecting milk samples from individual cows to prevent contamination with microorganisms present on the skin of udder and teats, on the hands of milking personnel and in the barn environment. During the sampling of raw milk directly from the udder, the udder and teats were cleaned and dried before sampling, and each teat end was scrubbed gently with cotton swabs moistened with 70% ethyl alcohol. Approximately 20 ml of pooled udder milk sample was collected from all functional teats of each cow into a sterile universal bottle after discarding the first few streams of milk. Bulk farm milk samples were taken aseptically after milk from all cows was collected into a bucket by thoroughly agitating the container. Samples were collected during dry and wet seasons, and sampling was performed during evening milking. Samples representing the wet season were collected from June to September, while samples for the dry season were collected from November to May, excluding short rainy season months (March and April). Sampling was interrupted when irregular rain occurred during the dry season.

Water (20 ml) that the farmers use to wash teats or hands was collected aseptically using sterile screw-capped universal bottles. Swab samples were collected from milking personnel’s hands, milking containers, bulking buckets and teats of the cows using sterile cotton-tipped swabs moistened in peptone water. The swab samples were taken before milking and immediately placed in to a sterile screw-capped tube containing 5 ml peptone water.

The samples were labelled with the farm code, sample type and date of collection with a permanent marker and transported to Asella Regional Veterinary Laboratory using an icebox for bacteriological analysis. The samples were stored at 4 °C and cultured the next morning (within 15–20 hours). Informed consent was obtained from each dairy farmer before sampling, and they were asked to rule out whether their cows had been given antibiotic treatment recently.

### Questionnaire survey

A structured questionnaire survey was conducted to assess factors that thought to influence the hygienic quality of bulk milk. Farm owners were interviewed on their personal information, herd size, whether dairying primary income, experience in dairying in years, training on hygienic milk production, use of warm water and/or detergent for milk utensil cleaning, use of warm water for teat washing, smoking of milk utensils, raw milk consumption habits and education level. When an activity had been performed by a hired laborer or a family member other than the farm owner, they were interviewed on that specific activity. For example, activities such as milking, and cleaning practices. Data on factors such as hygiene of the barn/cows/udder/milkers, hygiene of milk utensil, cow shade roof type, material of milk utensil, premilking udder preparation practices and milking procedures were collected by observation. The questionnaires were conducted face-to-face during the milking procedure. To avoid variation among individuals, the questionnaire survey was conducted by the first author of this paper (AD). The questions were originally written in English and translated into Afaan Oromo or Amharic languages when administered.

### Cow and udder hygiene scoring

The hygiene of the cows was evaluated based on the visual cleanliness scores described by Schreiner and Ruegg [39]. Evaluation was performed in the legs, flanks, abdomen, and udder of each animal. Score 1 (VC) indicates very clean, score 2 (C) indicates clean, score 3 (D) indicates dirty, and score 4 (VD) indicates very dirty. Farms were considered clean if the number of milking cows with cleanliness scores of VC and C were equal to or more than 50% of the total milking cows in the herd and taken as dirty if the number of milking cows with cleanliness scores of D and VD were equal to or more than 50% of the total milking cows in the herd. Udder scoring followed the scoring system described by Schreiner and Ruegg [39], where score 1 was free of dirt, score 2 was slightly dirty (2–10% of surface area), score 3 was moderately covered with dirt (10–30% surface area) and score 4 was covered with caked on dirt (> 30% of surface area).

### Enumeration of contaminating bacteria

Bulk farm milk samples were cultured to determine milk quality indicators such as TBC, CC, and CPS. Sample preparation was performed following the International Organization for Standardization protocol (ISO 8261:2001) [40]. Tenfold serial dilution of milk was performed by transferring 1 ml of milk of the previous dilution into 9 ml of 0.1% peptone water and was mixed by vortex. One milliliter of milk was discarded from the last dilution. Each sample was serially diluted up to 10^− 8^ for TBC and 10^− 6^ for CC and CPS.

TBC was enumerated on plate count agar (HiMedia Ltd., Mumbai, India) according to ISO 4833:2003 [41], while CC was counted on violet red bile agar (HiMedia Ltd., Mumbai, India) according to ISO 4832:2006 [42] using the pour plate method in both cases. One hundred microliters of serially diluted milk was aseptically withdrawn from each dilution using a micropipette and plated using 15–20 ml plate count agar and violet red bile agar, which were kept at 47 °C in a water bath. After thorough mixing by rotating, the plated samples were allowed to solidify and incubated aerobically at 30 °C for 48–72 hrs and 24 hrs for TBC and CC, respectively.

For enumeration of CPS (*Staphylococcus aureus* and other species), 100 μL of serially diluted milk sample was transferred to Baird-Parker agar supplemented with 20% egg yolk and 3.5% potassium tellurite (Oxoid Ltd., Basingstoke, England) and spread by a bent glass rod. The plates were incubated at 37 °C for 24 hrs and the positions of typical colonies were marked on the bottom of the plates. The plates were reincubated for an additional 24 hrs, and new typical colonies were marked. Two to five typical staphylococcal colonies were picked and a coagulase test was performed. Typical colonies are black or grey, shining, convex, and surrounded by a clear zone [44].

Two consecutive petri dishes with colony counts between 30 and 300 per plate were considered for TBC, while plates that contained 10–100 and 15–300 colonies were considered for CC and CPS, respectively. Then, the bacterial count in the respective original sample was expressed as the number of colony forming units per ml (cfu/ml) of samples according to ISO 7218:2007 [43].

### Isolation and identification of *Staphylococcus aureus*

Isolation and identification of *S. aureus* was performed according to ISO 6888-1:1999 [44] with minor modifications. Milk samples were enriched overnight in tryptone soya broth (HiMedia, India) at 37 °C to improve the recovery of injured cells (ISO 6888-3:2004) [45]. The swab samples were incubated in peptone water overnight at 37 °C. A loopful of culture was streaked on Baird-Parker agar supplemented with egg yolk and potassium tellurite and incubated at 37 °C for 24–48 hours. Up to five well-isolated typical colonies were picked and inoculated into nutrient broth (HiMedia, India) and incubated at 37 °C for 24 hrs. Then, the broth culture was plated onto nutrient agar plates and incubated at 37 °C for 24 hrs for purification and further identification. The presumptive *S. aureus* colonies were further identified based on Gram staining, mannitol fermentation (HiMedia, India), catalase activity, and coagulase activity using freeze-dried rabbit plasma (Santa Fe Drive, Lenexa, USA).

### Data analysis

Data from laboratory analysis and questionnaire surveys were entered into Microsoft Excel spread sheets. Statistical analysis was performed using STATA 16 (StataCorp, College Station, Texas, USA). Descriptive statistics and linear regression models were used for data analysis. Bacterial count data were normalized by log_10_ transformation. The normality of the log-transformed data was tested by the visual inspection of histograms. In CC data where there was no bacterial growth on some plates, transformation was performed by adding 1 to each count, i.e., log (CC + 1). Geometric mean, maximum and minimum values were determined for TBC, CC and CPS using transformed data. Correlations among milk quality indicators were assessed using the Spearman rank correlation coefficient. A linear mixed model was built to analyse the relationship of milk quality indicators and the occurrence of *S. aureus* in bulk milk with various risk factors. First, variables were checked for collinearity, and those found to be correlated with each other were entered into the final model separately. Univariable linear regression analysis was used to screen risk factors affecting TBC, CC and CPS, while univariable logistic regression was used to assess factors related to the occurence of *S. aureus* in farm bulk milk. Variables with *p* ≤ 0.25 in the univariable analysis were entered into the multivariable regression model. The final model was built using the backward elimination method. *P* ≤ 0.05 was considered statistically significant.

## Results

### Sociodemographic characteristics and hygiene practices

In this study, farm owners of the selected 50 farms were interviewed. In the majority of study farms, dairy cows were managed intensively (84%). Hand milking was practiced in all farms and cows were milked twice a day, in the morning and evening. Raw milk was consumed by 56% of dairy farmers interviewed. The majority of respondents (74%) did not receive training on hygienic milk production practices and 71.4% of these dairy farmers consumed raw milk. Udder drying using towels was practiced by only 14% of dairy farmers, and they used one towel for multiple cows. The majority of farmers (92%) used plastic containers for milking and milk storage, while 8% used stainless steel containers or Mazzican. Smoking of milk containers was practiced by the majority of dairy farmers (72%) in the study area. They fumigate milk containers by burning the stems of *Olea europaea* subsp. *cuspidata*, locally known as *ejersa*. In most farms (80%), milking was performed by a family member, while 20% of the farms used hired milkers. Except for one government farm, the other farms did not have a separate milking parlor. Although inadequate, all dairy farmers practiced teat washing without using detergent and flush their hands with the water that they used for teat cleaning. Some farmers moistened the teats by inserting their fingers into the milk when they became dry between milking. None of the farmers practiced pre- or postmilking antiseptic teat dipping, nor did they practice fore-stripping. Descriptive statistics for sociodemographic characteristics and hygiene factors are presented in Tables 2 and 3.

### Microbiological quality of bulk milk

The TBC in farm bulk milk varied from 2.91 log cfu/ml (8.18 × 10^2^) to 7.55 log cfu/ml (3.56 × 10^7^), with a geometric mean of 5.25 log cfu/ml (1.79 × 10^5^). CC varied from no coliform growth to 5.21 log cfu/ml (1.6 × 10^5^), with a geometric mean of 3.1 log cfu/ml (1.25 × 10^3^), while the CPS count varied from 1.26 log cfu/ml (1.8 × 10^1^) to 5.02 log cfu/ml (1.1 × 10^5^), with a geometric mean of 2.97 log cfu/ml (9.23 × 10^2^). Forty (90%) bulk milk samples had coliform growth. TBC was moderately correlated with CC (*r* = 0.5) but weakly correlated with CPS (r = 0.1). The correlation between CC and CPS was also weak (r = 0.12). According to the European Commission, the standard limits for raw cow's milk intended for direct human consumption are, < 10^5^ cfu/ml, < 10^2^ cfu/ml, and < 2 x10^3^ for TBC, CC, and *S. aureus* counts, respectively [46, 47]. Accordingly, 66% (n = 33), 88% (n = 44), and 32 (n = 16) of farms had TBC, CC and CPS counts, respectively, that exceeded the acceptable limits.

### Occurrence and sources of *Staphylococcus aureus* contamination in bulk milk

Overall, 24.9% (108/434) of the tested samples harbored *S. aureus*. *S. aureus* was detected in 21 (42%) farm bulk milk samples. Considering the potential sources of bulk milk contamination, the occurrence of *S. aureus* was higher in pooled udder milk (37.3%), followed by teat swabs (22.5%), milking personnel hand swabs (18%), bulk container swabs (16.7%), milking container swabs (14%), and water for cleaning udder and milker’s hands (10%). The difference in the occurrence of *S. aureus* in different sample types was statistically significant (*P* = 0.0001). The occurrence of *S. aureus* was significantly high in bulk milk compared to the contaminating sources. The likelihood of occurrence of *S. aureus* was 3.3, 2.7, and 1.33 times higher in bulk milk, pooled udder milk and teat swabs, respectively, than in human hand swabs. Logistic regression analysis of *S. aureus* occurrence in different sample types is presented in Table 1.

**Table 1:** Logistic regression analysis of *S. aureus* occurrence in different sample types

Sample type	Sample size	No. *S. aureus* positive	% positive (95% CI)	OR (95% CI)	*P*-value
Hand swabs	50	9	18 (9.5–31.5)	1	–
Bulk farm milk	50	21	42 (28.9–56.3)	3.3 (1.32–8.23)	0.011
Pooled udder milk	102	38	37.3 (28.3–47.1)	2.7 (1.18–6.18)	0.018
Water for cleaning teat and hands	50	5	10 (4.1–22.3)	0.51 (0.16–1.63)	0.255
Milking container swabs	50	7	14 (6.7–27)	0.74 (0.25–2.18)	0.586
Bulk container swabs	30	5	16.7 (6.8–35.3)	0.91 (0.27–3.03)	0.879
Teat swabs	102	23	22.5 (15.4–31.2)	1.33 (0.56–3.13)	0.519
Overall	434	108	24.9 (21–29.2)		

*No.* Number, *CI* Confidence interval, *OR* Odds ratio, *p*-value = 0.0001

### Univariable regression analysis of risk factors associated with milk quality indicators and occurrence of *S. aureus* in bulk milk

Univariable regression analysis was performed to screen factors associated with milk quality indicators (TBC, CC and CPS) and occurrence of *S. aureus* in bulk farm milk. Variables with *P* ≤ 0.25 in the univariable regression analysis were selected to be entered into the multivariable regression model. Univariable regression analysis of risk factors associated with TBC, CC, CPS and contamination of bulk milk by *S. aureus* is indicated in Tables 2 and 3.

**Table 2:** Univariable regression analysis of sociodemographic factors associated with TBC, CC, CPS and the occurrence of *S. aureus* in bulk farm milk

Variable	Category	No. of farms (%)	*p*-value			
			log_10_ TBC	log_10_ CC	log_10_ CPS	Occurrence of *S. aureus*
Age of farm owner (yr)	Continuous		0.036	–	–	0.032
Production system	Urban	36 (72)	–	0.011	0.139	–
	Peri-urban	14 (28)				
Dairying primary income	Yes	29 (58)	–	0.147	–	–
	No	21 (42)				
Sex of milker	Male	23 (46)	–	0.044	0.053	0.058
	Female	27 (54)				
Age of milker	Continuous		0.043	–	–	–
Cow shade roof type	Iron sheet	37 (74)	0.245	0.019	0.094	
	Plastic	5 (10)				
	Open	8 (16)				
Herd size	< 10	46 (92)	–	0.048	–	0.197
	> 10	4 (8)				
Season	Dry	30 (60)	0.000	0.022	0.200	0.079
	Wet	20 (40)				

*TB* Total bacterial count, *CC* Coliform count, *CPS* Coagulase-positive staphylococci count. Note: Linear regression was used when the outcome/dependent variable was continuous (bacterial count data), while logistic regression was used for categorical variables (*S. aureus* presence/absence). *P* ≤ 0.25 in the univariable regression analysis are shown in the table

**Table 3:** Univariable regression analysis of hygiene-related risk factors affecting TBC, CC, CPS and the occurrence of *S. aureus* in bulk farm milk

Variable	Category	No of farms (%)	*p*-value			
			log_10_ TBC	log_10_ CC	log_10_ CPS	Occurrence of *S. aureus*
Barn cleanliness	Clean	20 (40)	0.000	0.001	–	0.003
	Dirty	30 (60)				
Cow cleanliness	Clean	20 (40)	0.000	0.001	–	0.003
	Dirty	30 (60)				
Udder cleanliness	Clean	21 (42)	0.000	0.001	–	0.002
	Dirty	29 (58)				
Milker’s hygiene	Clean	31 (62)	0.001	0.002	–	–
	Dirty	19 (38)				
Milk utensil hygiene	Clean	31 (62)	0.001	0.002	0.238	0.004
	Dirty	19 (38)				
Water for milk utensils cleaning	Cold water	18 (36)	–	0.005	–	–
	Soap and cold water	11 (22)				
	Hot water	5 (10)				
	Soap and hot water	16 (32)				
Smoking of milk utensils	Yes	36 (72)	0.241	–	0.214	–
	No	14 (28)				
Water for teat and hand washing	Cold water	28 (56)	0.003	0.000	0.027	–
	Warm water	22 (44)				

*TBC* Total bacterial count, *CC* Coliform count, *CPS* Coagulase-positive staphylococci count. Note: Linear regression was used when the outcome/dependent variable was continuous (bacterial count data), while logistic regression was used for categorical variables (*S. aureus* presence/absence). *P* ≤ 0.25 in the univariable regression analysis are shown in the table

The univariable linear regression analysis showed that age of the farm owner (*P* = 0.036), age of the milker (*P* = 0.043), barn cleanliness (*P* = 0.000), cow cleanliness (*P* = 0.000), udder cleanliness (*P* = 0.000), milker’s personal hygiene (*P* = 0.001), milk utensil hygiene (*P* = 0.000), cow shade roof type (*P* = 0.245), season (*P* = 0.000), smoking of milk containers (*P* = 0.241), and the use of warm water for teat washing (*P* = 0.003) were significantly associated with TBC.

Coliform count was found to be associated with dairy production system (*P* = 0.011), dairy as a primary income source (*P* = 0.147), sex of the milker (*P* = 0.044), barn cleanliness (*P* = 0.001), cow cleanliness (*P* = 0.001), udder cleanliness (*P* = 001), milking personnel cleanliness (*P* = 0.002), milk utensil hygiene (*P* = 0.001), cow shade roof type (*P* = 0.019), herd size (*P* = 0.048), season (*P* = 0.022), use of hot water for milk utensil cleaning (*P* = 0.005), and use of warm water for teat washing (*P* = 0.000).

Predictors associated with CPS in univariable linear regression analysis were dairy production system (*P* = 0.139), sex of milker (*P* = 0.053), milk utensil hygiene (*P* = 0.238), cow shade roof type (*P* = 0.094), season (*P* = 0.2), milk container smoking (*P* = 0.214), use of warm water for teat washing (*P* = 0.027), and use of hired milker (*P* = 0.076).

Factors associated with the occurrence of *S. aureus* in farm bulk milk in the univariable logistic regression analysis were age of the farm owner (*P* = 0.032), sex of the milker (*P* = 0.058), barn cleanliness (*P* = 0.003), cow cleanliness (*P* = 0.003), udder cleanliness (*P* = 0.002), milk utensil hygiene (*P* = 0.004), herd size (*P* = 0.197) and season (*P* = 0.079).

Sex of farm owner, experience in dairying, education level, training on hygienic milk production, material of milk utensil and udder/teat drying were not related to TBC, CC, CPS or the presence of *S. aureus* in bulk milk in univariable regression analysis.

### Multivariable regression analysis of risk factors associated with milk quality indicators and occurrence of *S. aureus* in bulk milk

The normality of the log-transformed data was checked by visual inspection of the histograms, and there was no evidence of nonnormality observed. In the multivariable regression model, TBC was associated with barn cleanliness (*P* = 0.000), cow cleanliness (*P* = 0.000), udder cleanliness (*P* = 0.000) and season (*P* = 0.027). Coliform count was significantly associated with barn cleanliness (*P* = 0.035), cow cleanliness (*P* = 0.035), udder cleanliness (*P* = 0.044), use of warm water for teat washing (*P* = 0.001), and production system (*P* = 0.007). The use of warm water for teat washing (*P* = 0.027) was the only factor associated with the reduction in CPS count in the studied farms. In multiple logistic regression analysis, dirty barns (*P* = 0.003), dirty cows (*P* = 0.003), soiled udder and teats (*P* = 0.002) and sex of the milker (*P* = 0.043) were associated with *S. aureus* occurence in bulk milk. Barn cleanliness, cow cleanliness and udder cleanliness were entered into the multivariable regression model separately because of multicollinearity among them. The final model for factors influencing TBC, CC, CPS and the occurrence of *S. aureus* in bulk farm milk is presented in Table 4.

**Table 4:** Multivariable regression analysis of factors associated with TBC, CC, CPS and contamination of bulk milk by *S. aureus*

Variable	Category	No of farms (%)	*p*-value			
			log_10_ TBC	log_10_ CC	log_10_ CPS	Occurrence of *S. aureus*
Barn cleanliness	Clean	20 (40)	0.000	0.035	–	0.003
	Dirty	30 (60)				
Cow cleanliness	Clean	20 (40)	0.000	0.035	–	0.003
	Dirty	30 (60)				
Udder cleanliness	Clean	21 (42)	0.000	0.044	–	0.002
	Dirty	29 (58)				
Season	Dry	30 (60)	0.027	–	–	–
	Wet	20 (40)				
Sex of milker	Male	23 (46)	–	–	–	0.043
	Female	27 (54)				
Production system	Urban	36 (72)	–	0.007	–	–
	Peri-urban	14 (28)				
Water for teat and hand washing	Cold	28 (56)	–	0.000	0.027	–
	Warm	22 (44)				

*TBC* Total bacterial count, *CC* Coliform count, *CPS* Coagulase-positive staphylococci count. Multivariable linear regression was used when the outcome/dependent variable was continuous (bacterial count data), while multiple logistic regression analysis was used in categorical variable (presence/absence of *S. aureus*). *P* ≤ 0.05 in the final model are shown in the table

## Discussion

In this study, the geometric mean of TBC in bulk milk in the studied farms was 5.25 log cfu/ml. Thirty three (66%) farms had TBCs that exceeded the acceptable limit for raw cow's milk intended for direct human consumption, which is < 10^5^ cfu/ml [46]. This indicates high food safety risk, as raw milk consumption is common in Ethiopia [29–31]. The relatively higher TBC in the present study might be linked to dirty barns and cows, soiled udders and teats, poor milking hygiene, unhygienic milking utensils, and poor personal hygiene, which were observed during the farm visits [2, 9, 15]. The increased TBC could also be attributed to mastitis [48]. The drainage system was poor in the majority of the farms in the study area resulting in wet floors especially during the rainy season. Faeces were not regularly removed. These conditions might have been resulted in soiling of the udder and teats, which might ultimately contaminate milk [49]. Udder and teat hygiene are the major factors associated with microbial contamination in bulk milk [15, 50]. Pre- and/or postmilking teat disinfection were reported to reduce the bacterial load on teat skin and were found to be the most effective practice against environmental bacteria [8, 51]. However, no pre- or postmilking teat disinfection was practiced in the study area, which may explain the high TBC in our study. Training of farmers on adoption of good milk production practices can improve the quality of milk by decreasing bacterial counts [52]. However, the majority of farmers in Asella area were not trained on hygienic milk production practices, which might play a role in increasing the bacterial load of bulk milk. Previous studies conducted in other parts of Ethiopia [53–55], Myanmar [56], Sri Lanka [57], India [58], Nigeria [59] and Burkina Faso [60] reported mean TBC counts higher than our finding. In contrast, lower mean TBC counts were reported in Gondar, Ethiopia [61], Tanzania [62], the USA [13, 17, 63, 64], Belgium [8], Italy [10] and Ireland [49]. A study conducted in Brazil [15] and Chile [65] reported TBC values comparable to the present finding. The variation with previous works could be due to differences in management and hygiene practices, farm size and agro-ecology [8, 19]. The fact that in this study raw milk was sampled immediately after milk from all cows bulked in to a container might have contributed to the variation.

Consistent with our findings, previous studies associated dirty barns [16, 49], dirty cows [16, 56], and soiled udder and teats [7, 15, 61] with elevated TBCs in bulk milk. In the present study, season significantly associated with TBC, with higher counts during the wet season than during the dry season. This agrees with previous reports [8, 19, 66]. During rainy seasons, the udder and teats of dairy cows are soiled with feces and mud, which increase microbial contamination of milk [8]. Premilking udder preparation is essential to produce high-quality milk [5]. A teat cleaning procedure that includes wet cleaning followed by manual drying with a towel reduces microbial contamination in milk [7, 51]. In this study, herd size did not influence TBC, which agrees with reports from Myanmar [56] and the USA [17]. The absence of an association between herd size and TBC in this study could be attributed to small differences in the number of cows among the farms.

Total coliform counts > 10^3^ cfu/ml are indicative of hygiene-related problems in milk production [15]. The geometric mean of CC in this study was 3.1 log cfu/ml, which was higher than the standard set by the EC for raw milk intended for direct human consumption, which is < 100 cfu/ml [46]. Bulk milk samples from 40 (90%) farms had coliform growths and 88% of farms had CC counts higher than this limit. This indicates possible contamination by pathogenic bacteria of public health significance, such as *E. coli* O157:H7 [15, 19], as people prefer raw milk consumption in Ethiopia [29–31]. The relative increase in CC in the present study could be attributed to poor farm environment hygiene and dirty cows and udders, which were evidenced during the farm visits [13, 17]. A high CC is an indicator of faecal contamination from dirty udders and teats [17]. High CC can also denote improper cleaning of milk equipment, and coliform growth in milk residues in milk equipment [10], and poor quality of water used for cleaning [17]. Coliforms are important environmental mastitis pathogens [13], and the primary sources of exposure are the presence of moisture, mud and manure in the environment of the cow [39]. Mastitis was reported to be prevalent in the study area [67]. The geometric mean of CC in the present study was relatively low compared with reports from other parts of Ethiopia [54, 55, 68], Sri Lanka [57] and Myanmar [56]. However, our result was relatively higher than the reports from the USA [13, 17, 64], Belgium [8], Italy [10] and Nigeria [59]. The CC in our finding agrees with reports from central Ethiopia [53] and Brazil [15].

In line with our findings, dirty barns [50], dirty cows [5, 56] and soiled udder and teats [16] were reported to be associated with increased CC in bulk milk. In this study, the use of warm water to clean teats was related to low CC. Similarly, a study conducted in Irish dairy herds reported an association between the use of heated water in the milking parlor and low bacterial counts [49]. The use of hot water is indicated to enhance washing efficacy [10]. CC is found to be significantly higher in peri-urban areas than in urban areas. This could be attributed to soil barn floor that can easily get muddy and low awareness on hygienic milk production practices.

The correlation between TBC and CC (r = 0.5) in this study corroborated previous studies that reported a moderate to high correlation [13, 15, 56, 69]. This indicates that TBC could be used as a single microbiological hygienic indicator for total aerobic bacteria present in raw milk [15].

The geometric mean of CPS in bulk milk in the present study was 2.97 log cfu/ml, which is with in the EC limit for raw cow's milk. Raw cow’s milk intended for direct human consumption must have *S. aureus* count of < 2,000 cfu/ml, which makes milk in 32% of studied farms unsafe for raw consumption [47]. This may present a risk for staphylococcal food poisoning, as raw milk is widely consumed in Ethiopia [29–31]. Nearly comparable CPS was reported in Greece [70], while a higher count was reported in other parts of Ethiopia [55, 71, 72] and Brazil [15]. However, lower counts were reported from Ireland [49] and Portugal [73]. In the final model, CPS was significantly higher in teats cleaned with cold water than in those cleaned with warm water, and this agrees with a previous report [49]. Adequate premilking teat sanitation reduces the load of bacteria on teats and ensures the production of high-quality milk [7, 12].

The result of this study showed that the occurrence of *S. aureus* in bulk milk (42%) was high compared to previous reports from other parts of Ethiopia [26, 28, 54, 74], Uganda [75], Brazil [76], the USA [17] and China [77]. Our finding complies with previous reports from Ethiopia [27], Greece [70] and Italy [78]. In contrast, slightly higher *S. aureus*-occurrences of 66, 61, 54, 51, 48% were reported in Brazil [15], India [58], Portugal [73], Ireland [49] and the USA [79], respectively. The high occurrence of *S. aureus* in this study is concerning because many strains can produce enterotoxins and consequently cause food-borne intoxication in consumers [32]. The high occurrence of *S. aureus* in bulk milk in this study could be indicative of mastitis in the studied farms. *S. aureus* might have also contaminated bulk milk from human handlers, milk utensils, the environment, and the udder and teat skin of dairy cows [78]. The absence of premilking teat preparation is a risk factor for the presence of *S. aureus* in bulk milk [15]. Thus, it is important that mastitis is prevented and hygienic milk production practices are implemented to safeguard the public from staphylococcal food poisoning [78].

In this study, pooled udder milk (37.3%) was more contaminated by *S. aureus* than teat swabs (22.5%), milking personnel hand swabs (18%), bulk container swabs (16.7%), milking container swabs (14%) and water used for cleaning udder and hands of milking personnel (10%). This result indicates that these could be potential sources for contamination of bulk milk. The widespread isolation of *S. aureus* from bulk milk and potential contaminating sources indicates poor management, poor hygiene practices or udder infections in the studied farms. Compared to the 37.3% isolation rate in our study, lower *S. aureus* udder milk contaminations of 19.6% [29] and 15.3% [80] were reported in Ethiopia, while a much lower rate of 5.5% was recorded in Brazil [76]. The high occurrence of *S. aureus* in udder milk in this study could be associated with mastitis [22]. In Ethiopia, *S. aureus* contamination rates of 32 and 11.1% were reported in milkers’ hand swabs and milking bucket swabs, respectively [29]. Compared to our findings, a study conducted in the North Shewa zone of Ethiopia reported slightly higher *S. aureus* contamination rates of 25 and 20% in hand and bucket swab samples, respectively [80]. In Brazil, a relatively lower *S. aureus* contamination rates of 3.3 and 3.6% were reported in swab samples from milkers’ hands and milk utensils, respectively [76]. In Algeria, water used for cleaning during milking (50.9%) was more contaminated by *S. aureus* than swab samples from the hands of milkers (39.6%), udder (28.9%) and milk utensils (5.7%) [81]. The variation in the occurrence of *S. aureus* with previous findings might be attributed to differences in agroecology, management, hygiene practices on the farm, and/or the sensitivity of the detection methods used.

The identification of farm-level risk factors affecting the occurrence of *S. aureus* in bulk milk is important in the prevention and control of the organism [79]. Risk factors that affected the presence of *S. aureus* in bulk milk in the study area were barn cleanliness, cow cleanliness, udder cleanliness and sex of the milker. In line with our findings, *S. aureus* was reported to be more prevalent in farms with poor milking hygiene [82] and dirty udders [39] than in those with better hygiene. In this study, the occurrence of *S. aureus* was higher in farms where milking was performed by males than in those milked by female farmers. This might be attributed to the better experience of females in cleaning. In Ethiopia, milking of cows and, cleaning of milk utensils and the barn are mostly performed by females.

More than half (56%) of the farmers interviewed in this study consumed raw milk, which could be due to a lack of awareness, as raw milk consumption was higher in farmers who did not receive training. In previous reports, raw milk was consumed by 35% of dairy farmers in Ethiopia [29], 42.3% in the USA [83] and 65% in Tanzania [62]. According to a review [84], 35–60% of farm families and farm employees consume raw milk. Raw milk consumption is not generally encouraged because even the most appropriate hygienic procedures do not always ensure the absence of the pathogens [2]. In this study, only 26% of farm owners had received training on quality milk production practices. Lack of training on hygienic milk production practices increases the risk of milk contamination at the farm level [4]. Similar to other previous studies conducted in different parts of Ethiopia [29, 61, 68, 71], the majority of farmers (92%) in the study area used plastic containers for milking and milk storage. This could affect milk quality, as plastic containers scratch easily and provide hiding places for the growth of bacteria [4, 29, 61]. They are also sensitive to heat, allowing the multiplication of microorganisms during milk handling. Aluminum and stainless steel equipment are preferred for milk handling [4]. Although inadequate, all the dairy farmers interviewed were observed to practice teat washing and flush their hands with the water that they used for teat cleaning. However, only 14% of dairy farmers practiced udder drying, and they used a common towel for multiple cows, which can increase milk contamination and the incidence of mastitis [7]. Slightly lower proportions of farmers (52–80%) were reported to wash their hands before milking in pastoral and agro-pastoral areas of eastern Ethiopia [85]. In and around Gondar, 75 and 68.3% of dairy farmers were reported to wash udder and their hands before milking, respectively [61]. Cleaning teats and manual drying with towels reduce the microbial contamination in milk [7]. This is because water laden with bacteria on the udder and teats can enter milking equipment and increase bacterial contamination of milk [7]. Failure to wash hands adequately exposes milk to bacterial contamination [68]. During milking, some dairy farmers were observed to moisten the teats by inserting their fingers into the milk when the teats become dry between milking, which may introduce microorganisms into the milk [4]. Pre- and/or postdipping improves the microbiological quality of milk by reducing the teat surface microbial load [5]. Fore-stripping prevents contaminated milk from entering the dairy chain and is also used to check for clinical mastitis [5]. However, the farmers in the study area neither practiced pre- or postmilking antiseptic teat dipping, nor did they practice fore-stripping.

### Limitations of the study

This study was limited by sample size and study area. Replication of sampling seasons is necessary to increase the accuracy of the results. Therefore, the present findings encourage further investigations using a large sample size covering a wide study area, replication of sampling seasons and molecular characterization of *S. aureus* isolates.

## Conclusions

The findings of the present study revealed low-quality bulk milk with TBC, CC and CPS counts higher than acceptable limits. Moreover, *S. aureus* was widely distributed in bulk milk and its potential contaminating sources. This may present a public health hazard, as raw milk is widely consumed in Ethiopia. Dirty barns and cows and soiled udder and teats were identified as risk factors affecting TBC, CC and the contamination of farm bulk milk by *S. aureus*. TBC was found to be associated with the rainy season. CC and CPS were significantly decreased by the use of warm water to clean teats. The questionnaire survey result showed widespred raw milk consumption habit, low level of training and poor hygienic milking practices. This study suggests awareness creation to dairy farmers and the public on hygienic milk production and heat treatment of milk before consumption.

## Data Availability

The datasets used and/or analysed during the current study are available from the corresponding author on reasonable request.

## Abbreviations

CFU: Colony forming units
CC: Coliform count
TBC: Total bacterial count
CPS: Coagulase-positive staphylococci count
EC: European Commission
ISO: International Organization for Standardization
CI: Confidence interval
OR: Odds ratio
